# Rapid-cycle deliberate practice versus after-event debriefing clinical simulation in cardiopulmonary resuscitation: a cluster randomized trial

**DOI:** 10.1186/s41077-022-00239-8

**Published:** 2022-12-28

**Authors:** Leandro Teixeira de Castro, Andreia Melo Coriolano, Karina Burckart, Mislane Bezerra Soares, Tarso Augusto Duenhas Accorsi, Vitor Emer Egypto Rosa, Antônio Sérgio de Santis Andrade Lopes, Thomaz Bittencourt Couto

**Affiliations:** 1 Centro de Simulação Realística, Hospital Israelita Albert Einstein, Avenida Albert Einstein, 627, São Paulo, SP 05652-900 Brazil; 2 Instituto Israelita de Ensino E Pesquisa, Hospital Israelita Albert Einstein, São Paulo, Brazil

**Keywords:** Cardiopulmonary resuscitation, Time-to-treatment, Defibrillator, Medical education

## Abstract

**Introduction:**

Rapid-cycle deliberate practice (RCDP) is a simulation-based educational strategy that consists of repeating a simulation scenario a number of times to acquire a planned competency. When the objective of a cycle is achieved, a new cycle initiates with increased skill complexity. There have been no previous randomized studies comparing after-event debriefing clinical manikin-based simulation to RCDP in adult cardiopulmonary resuscitation (CPR).

**Methods:**

We invited physicians from the post-graduate program on Emergency Medicine of the Hospital Israelita Albert Einstein. Groups were randomized 1:1 to RCDP or after-event debriefing simulation prior to the first station of CPR training. During the first 5 min of the pre-intervention scenario, both groups participated in a simulated case of an out-of-hospital cardiac arrest without facilitator interference; after the first 5 min, each scenario was then facilitated according to group allocation (RCDP or after-event debriefing). In a second scenario of CPR later in the day with the same participants, there was no facilitator intervention, and the planned outcomes were evaluated. The primary outcome was the chest compression fraction during CPR in the post-intervention scenario. Secondary outcomes comprised time for recognition of the cardiac arrest, time for first verbalization of the cardiac arrest initial rhythm, time for first defibrillation, and mean pre-defibrillation pause.

**Results:**

We analyzed data of three courses conducted between June 2018 and July 2019, with 76 participants divided into 9 teams. Each team had a median of 8 participants. In the post-intervention scenario, the RCDP teams had a significantly higher chest compression fraction than the after-event debriefing group (80.0% vs 63.6%; *p* = 0.036). The RCDP group also demonstrated a significantly lower time between recognition of the rhythm and defibrillation (6 vs 25 s; *p* value = 0.036).

**Conclusion:**

RCDP simulation strategy is associated with significantly higher manikin chest compression fraction during CPR when compared to an after-event debriefing simulation.

**Supplementary Information:**

The online version contains supplementary material available at 10.1186/s41077-022-00239-8.

## Introduction

Survival after cardiac arrest (CA) has increased slowly throughout the last decade. A recent systematic review found a global rate of return of spontaneous circulation of 29.7% after CA, with survival to hospital discharge of 8.8% [1]. In the USA, the survival rate after discharge from out-of-hospital CA is about 10.6% [2]. Despite the advances in the science of cardiopulmonary resuscitation (CPR), there is a large disparity in outcomes of CA, with different results depending on the time of the day and locality of the CA [1, 3, 4]. In addition, even with common protocols and standardized courses, there are several disparities in the individual performance of healthcare professionals, which result in diverse clinical outcomes after CPR [5]. For this reason, developing better methods for CPR training is crucial to improving survival from CA. Two topics identified by the American Heart Association concerning the need for further studies on education in resuscitation are the study of educational strategies in CPR and the use of repetitive practice and mastery learning [5].

Clinical simulation in the emergency setting is commonly performed by letting students manage a clinical case for a determined period of time with little to no interference by the facilitator; this experience is then followed by a debriefing session [6]. In rapid-cycle deliberate practice (RCDP), a strategy of simulation described by Hunt in 2014 [7], the same clinical case is divided into smaller cycles with predetermined goals; students participate in each cycle a number of times until all the goals for that cycle are met.

Although studies have shown good performance in resuscitation efforts of teams after RCDP was applied [7–9], as well as in simulated scenarios of intra-hospital CPR compared to the standard course of basic life support (BLS) [10, 11], so far there have been no studies comparing the after-event debriefing method of clinical simulation versus rapid-cycle deliberate practice for advanced CPR training in adults.

We hypothesized that clinical simulation with RCDP in a scenario of cardiac arrest in adults would lead to better outcomes in key measures of CPR quality than with an after-event debriefing simulation strategy.

## Methods

This was a cluster randomized trial controlled by the standard intervention (after-event debriefing). The study was conducted at the Simulation Center of the Hospital Israelita Albert Einstein, Sao Paulo, Brazil. The trial protocol was approved by the institutional ethics board with registration number CAAE: 8840.4418.5.00000.0071.

### Participants

We invited physicians enrolled in the Emergency Medicine post-graduate Course of the Hospital Israelita Albert Einstein to participate. This is a specialization course in emergency medicine for physicians that includes theoretical activities, supervised observation of clinical encounters, and clinical simulation. The course curriculum also provides theoretical training in emergency cardiovascular care, including a 4-h case-based discussion regarding the management of cardiac arrest in the emergency room. On the day of the clinical simulation, an 8-h-long course is held at the Simulation Center.

Recruitment for the study occurred in the dates when clinical simulations were scheduled. Physicians were informed about the objectives of the study and were invited to participate after providing written consent.

### Trial procedures

Participants were divided randomly into groups that were maintained the same for all activities. Randomization of groups and intervention occurred in a CA scenario held during the morning. Immediately before the scenario, groups were randomized 1:1 to participate in RCDP (intervention group) or after-event debriefing simulation (control group). The randomization process involved the use of sequentially numbered, opaque, sealed envelopes previously sorted by an independent third party (facilitator or analyst not involved with the scenario). During the first 5 min of the scenario, both groups attended a simulated case of out-of-hospital CA in an adult manikin without interference by the facilitator; after the first 5 min, each scenario was then facilitated according to group allocation (RCDP or after-event debriefing). In the control arm, participants continued to provide resuscitation efforts without facilitator interference for a total of 10 min, followed by 30 min of debriefing using the PEARLS method [12].

#### Debriefing

Facilitators began the analysis phase of debriefing in the control arm by asking for a brief self-assessment by the participants. They then provided feedback making sure that the main learning points for the scenario were discussed (as described in Additional file 1), by using directive feedback or an advocacy-inquiry approach at the instructors’ discretion.

In the RCDP arm, the scenario was restarted after the first 5 min. When errors occurred, the facilitator interfered immediately, providing brief feedback and prompting the participants to resume or restart the efforts up to the end of the cycle, according to the strategic principles of RCDP (objectives of each cycle described in Additional file 1).

The total scenario duration was 40 min in both arms. We used the RessusciAnne® simulator with remote control as our manikin and the Zoll R series® manual defibrillator in all simulations. Participants’ roles in the teams were self-assigned in all scenarios.

Facilitators in this course were two physicians with more than 4 years of experience with clinical simulation (including RCDP) and worked as instructors of the Clinical Simulation Center for at least 20 h a month (approximately 50 simulation activities every year). They also received continuing medical education regarding simulation training for approximately 30 h a year.

#### Outcome assessment

All groups participated in a second scenario of out-of-hospital CPR in the afternoon with 10 min of duration without intervention by the facilitator—this scenario was used for outcome assessment. All other scenarios were performed as planned by the course curriculum, with no interference from this study; these scenarios were applied in the exact same way to all of the participants.

The primary outcome for this study was the chest compression fraction during CA in the post-intervention scenario; chest compression fraction was defined as the time in which chest compressions were being performed divided by the time in which the manikin had no pulse after the initial pulse check. Secondary outcomes included time for recognition of the cardiac arrest since the beginning of the scenario, time for first verbalization of the cardiac arrest initial rhythm (since the moment when pads were placed), time for the first defibrillation (since confirmation of an absent pulse), and mean pre-defibrillation pause (time between the last chest compression before rhythm check and defibrillation).

#### Data collection

Data collection was performed through video recordings of every simulation by the Simulation Center’s cameras and audio equipment. The video files were then renamed using prespecified nomenclature and sent to two of the study investigators for analysis; both investigators had more than 3 years of experience with clinical simulation and were practicing physicians in the emergency department. During the first video analysis, besides coding of the number of participants in the group, the following events were time coded: beginning of the scenario, verbalization of an absent central pulse, first appearance of a cardiac rhythm in the defibrillator monitor, first rhythm verbalization, every chest compression pause for rhythm check, every defibrillation, and the ending of the scenario. On a second analysis, the beginning and ending of every chest compression cycle were time coded.

### Statistical analysis

We calculated that a sample size of 6 groups in each arm (8 participants in each group) would provide greater than 80% power to detect a difference of 20% in the primary outcome, using a two-sided significance level of 0.05.

All continuous variables with non-normal distribution are presented as medians and interquartile intervals and compared using the Mann–Whitney test. Categorical variables are presented as proportions and compared using the chi-square test. Statistical analyses were performed with Statistical Package for the Social Sciences (SPSS) v.26.0 (IBM Corp, 2019).

## Results

**Fig. 1:**
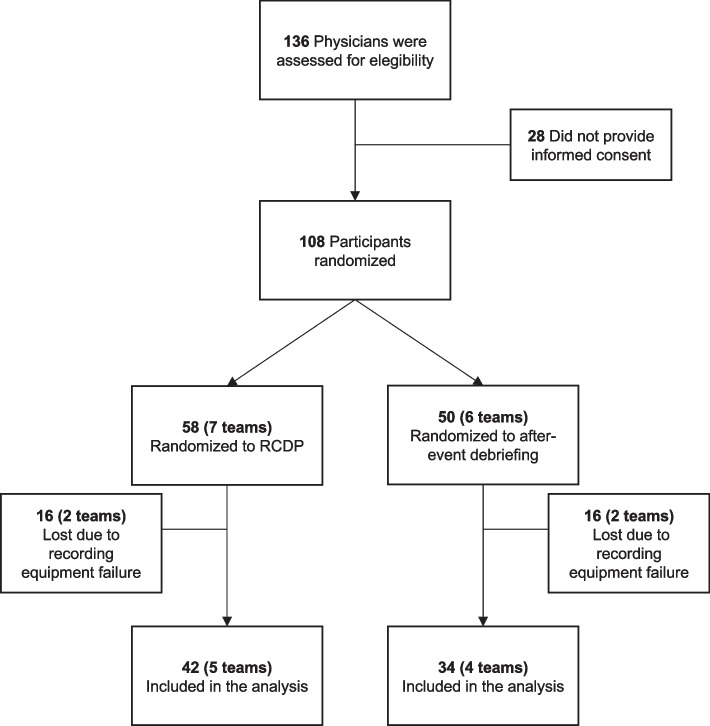
Study design flowchart

We invited 136 physicians to participate in the study on four different dates between June 2018 and July 2019. Of these, 108 accepted to participate and signed the consent term. Participants were divided into teams and randomized 1:1 for RCDP or standard intervention (six teams randomized for standard intervention and seven teams for RCDP intervention). Because of failures in the recording equipment in one of the dates, four groups composed by 32 students were lost in the analysis (two for each intervention). We evaluated data from the 76 remaining students, divided into nine teams (four in the standard intervention group and five in the RCDP group) (Fig. 1).

Each team had a median of 8 students (interquartile interval = 1). Outcomes observed in each of the arms and comparisons between them are presented in Table 1.

**Table 1:** Outcome comparison between arms

Outcomes	After-event debriefing (*n* = 4 teams)		Rapid-cycle deliberate practice (*n* = 5 teams)		*p*-value
Chest compression fraction	0.636	0.056	0.800	0.055	0.027
Time to first defibrillation^a^	47	8	10	8	0.014
Median pre-defibrillation pause^a^	25	15	6	6	0.036
Time until cardiac arrest recognition^a^	21	27	15	4	0.104
Time until rhythm is verbalized^a^	15	8	9	5	0.266

All outcomes are presented as median and interquartile ranges

^a^Measured in seconds

**Fig. 2:**
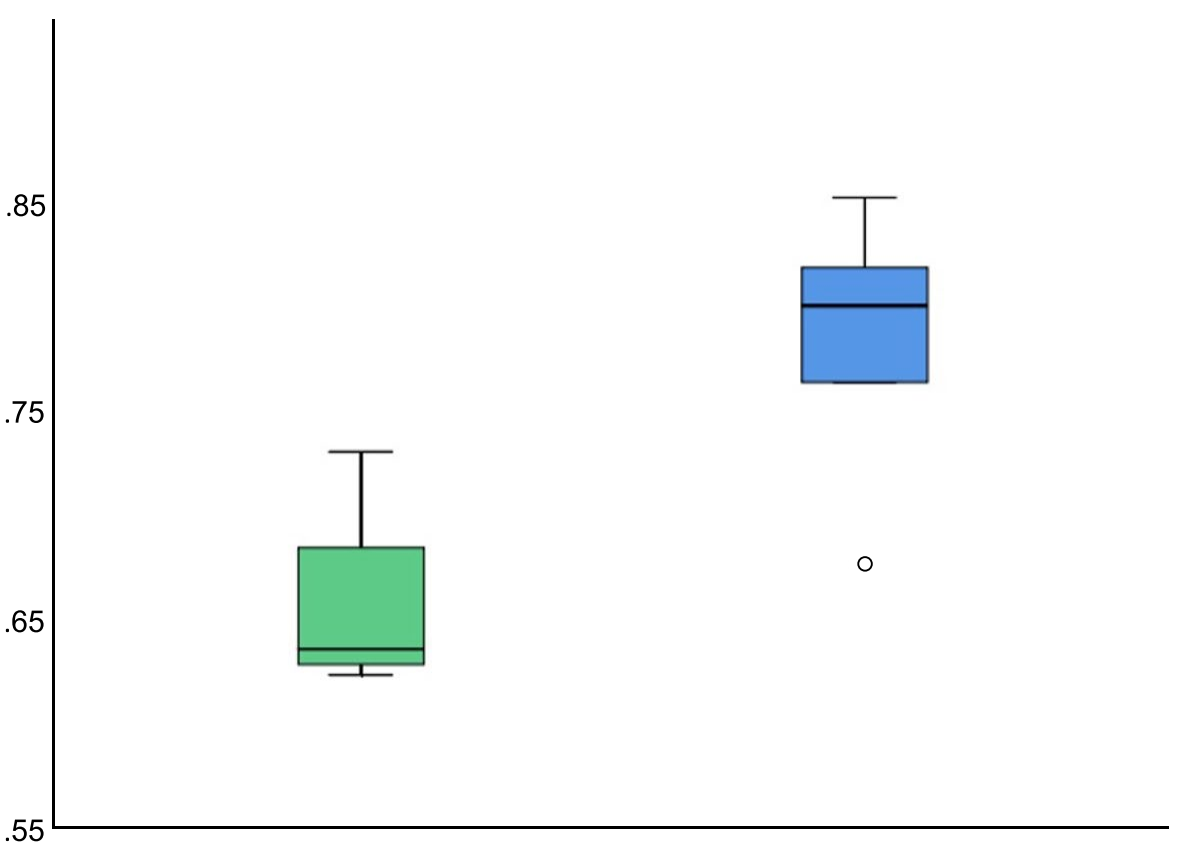
Chest compression fraction comparison between arms

Groups randomized to RCDP had a greater proportion of chest compression fraction (median = 0.800 vs 0.636; *p-*value = 0.027), as shown in Fig. 2. The RCDP arm also had a lower time to first defibrillation (median = 10 vs 47 s; *p*-value = 0.014) and lower median pre-defibrillation pause (6 vs 25 s; *p*-value = 0.036). Other CPR metrics were not significantly different between the arms.

## Discussion

In this cluster randomized trial, the RCDP strategy was associated with significant improvement in chest compression fraction during CPR in a manikin-simulated environment, when compared to after-event debriefing.

One way to explain the learning during clinical simulation is throughout the Kolb Experiential Learning Cycle [13]. Participants experience a concrete situation, a simulated case, and they have the opportunity to identify performance gaps in which they can reflect on [6]. During debriefing, they have the opportunity to reflect on the simulation and their performance guided by a facilitator. This model can be ideal for a critical discussion of complex cases with multiple possible outcomes, but also brings a number of important limitations, among them the impossibility of participants to practice again after receiving feedback; hands-on practice time is also reduced because of the allocated time for the debriefing section [14]. In rapid-cycle deliberate practice, the time in which students actively participate in deliberate practice is maximized, with multiple opportunities to practice skills in the correct form. This also enables students to accept direct feedback without adopting a defensive posture, given that it is offered continuously and divided in smaller breaks throughout the case [7]. In our study, CPR quality and early defibrillation were emphasized as specific goals to be met during the cycles performed by the RCDP group; this group reached significantly better outcomes in these areas compared to the standard intervention group. As multiple studies have already established a clear survival benefit associated with early defibrillation [15–18] and high-quality CPR [19, 20] in out-of-hospital CA, our findings suggest that we may improve patient-oriented outcomes with RCDP training.

Since RCDP was introduced, many of the studies regarding its efficacy have been focused on the pediatric population. In a single-blind trial published in 2019, randomized participants had a better performance in choreography to conduct pediatric orotracheal intubation [21]. Another study showed an increase in knowledge gain in sepsis care in the pediatric population [22]. This strategy increased the trust of medical residents in their skills in providing pediatric trauma care [23]. Another trial showed increased performance related to human factors during pediatric resuscitation [8].

Our results follow the trend of previously published studies in this area by expanding the findings to simulations in adult resuscitation. Assessment of outcomes on the same day of training enabled us to limit the number of potential confounders for the differences of performance among the groups. Because all participants were students from the same specialization course, with prior training in the theoretical aspects of resuscitation, the risk of significant baseline differences in knowledge was also minimized.

This study has some limitations. Due to issues regarding audio and video caption, four groups (two in the intervention arm and two in the control arm—32 participants in total) could not have their outcomes assessed; we could not determine if the performance of these groups could have affected the final results. Another limitation was the impossibility to conceal the simulation strategy, given that both facilitator and participants necessarily knew what type of simulation was being trained. Assessment of outcomes was also not blinded, given that assessors had access to the full content of the videos recorded throughout the course. We evaluated only aspects related to time of actions of participants and not necessarily related to the quality of these actions; there is the possibility that teams were faster without necessarily having better performance (like chest compression depth and full recoil)—this is probably not likely, considering that many metrics of efficient resuscitation are related to the time for each intervention. We also did not have sociodemographic data and level of expertise at baseline for the participants; even though every participant was randomly allocated to each of the groups before the beginning of the simulations, the absence of this baseline data could be a potential source of bias in our study.

Finally, we only evaluated the performance of each team concerning their skills on resuscitation immediately after the course. Retention and translation of these skills to the clinical environment are desirable metrics in the assessment of any evaluation for any problem in teaching [24], but in our study, these metrics were impossible to be measured, given that our participants worked in different institutions.

## Conclusion

In conclusion, this study showed that the RCDP strategy is associated with better performance of resuscitation teams in critical actions during care for CA in adults and should be considered as an option for the training of teams involved in emergency cardiovascular care. Future studies may further explore the effectiveness of RCPD strategy versus formal advanced cardiovascular life support training and if different simulation strategies may impact patient-oriented outcomes in CA care.

## Supplementary Information

**Additional file 1.** Clinical scenario.

## Data Availability

The data that support the findings of this study are available from the corresponding author, LTC, upon reasonable request.

## Abbreviations

BLS: Basic life support
CA: Cardiac arrest
CPR: Cardiopulmonary resuscitation
RCDP: Rapid-cycle deliberate practice
