# Supplementary material for: Rapid-cycle deliberate practice versus after-event debriefing clinical simulation in cardiopulmonary resuscitation: a cluster randomized trial
Source: Adv Simul (Lond). 2022 Dec 28;7:43. doi: 10.1186/s41077-022-00239-8 (PMC9798613; doi:10.1186/s41077-022-00239-8)
Supplement: Supplementary file 1 — Additional file 1. Clinical scenario. [file 41077_2022_239_MOESM1_ESM.docx]

**APPENDIX 1**

CLINICAL SCENARIO - RCDP

This scenario must be facilitated using the Rapid Cycle Deliberate Practice model. Each group of four participants will be responsible for performing all the actions necessary to meet each cycle’s goals. In case of any critical error, the facilitator must interrupt the scenario and provide objective feedback. The simulation is then immediately resumed or reinitiated according to the discretion of the facilitator.

Each cycle must be repeated until the facilitator feels confident that all participants performed in a way that met all the goals intended for that cycle.

RessusciAnne® simulator must be set to the rhythm “Ventricular fibrillation”.

Clinical setting for all cycles: “You are in the hospital’s Emergency Room. A 30-year-old male is brought by the Emergency Medical Service with loss of conscience”.

Objectives for each cycle:

CYCLE 1

- Main objective: practice the sequence of initial recognition and management of a cardiac arrest patient
  - check responsiveness
  - call for help with defibrillator
  - assess for presence of breathing and pulse
  - initiate high-quality chest compressions
    - 100-120 compressions/minute
    - 5-6cm deep
    - letting complete chest recoil
    - no interruptions longer than 10 seconds
  - establish leadership
  - assign roles for each member of the team

CYCLE 2

- Main objective: ensure high quality ventilation
  - maintain objectives described in Cycle 1
  - high-quality ventilation
    - optimal mask positioning (C:E technique)
    - two one-second ventilations for each 30 compressions
    - no air leaks through the mask

CYCLE 3

- Main objective: early appropriate defibrillation
  - maintain objectives described in Cycles 1 and 2
  - early defibrillation
    - turn defibrillator on defibrillation mode
    - confirm that team members are clear of any patient contact
    - provide a clear rhythm diagnosis
    - defibrillate in under 10 seconds from rhythm diagnosis
    - resume chest compressions immediately after defibrillation

Materials needed for this scenario:

- RessusciAnne® simulator with remote control
- Zoll R series® defibrillator
- Bag-valve-mask device with reservoir
- Laryngoscope
- Endotracheal tubes (size 7.0, 7.5 and 8.0)

CLINICAL SCENARIO - CONTROL GROUP

This scenario must be facilitated using the traditional model (clinical simulation followed by debriefing). One group of four participants will be responsible for performing all the actions required in the scenario for a total of 10 minutes, followed by a 30-minute debriefing section.

RessusciAnne® simulator must be set to the rhythm “Ventricular fibrillation”.

Clinical setting: “You are in the hospital’s Emergency Room. A 30-year-old male is brought by the Emergency Medical Service with loss of conscience”.

Main points to be discussed during debriefing:

- Initial recognition and management of a cardiac arrest patient
  - check responsiveness
  - call for help with defibrillator
  - assess for presence of breathing and pulse
  - initiate high-quality chest compressions
    - 100-120 compressions/minute
    - 5-6cm deep
    - letting complete chest recoil
    - no interruptions longer than 10 seconds
  - establish leadership
  - assign roles for each member of the team
- High quality ventilation
  - optimal mask positioning (C:E technique)
    - two one-second ventilations for each 30 compressions
    - no air leaks through the mask
- Early appropriate defibrillation
  - turn defibrillator on defibrillation mode
    - confirm that team members are clear of any patient contact
    - provide a clear rhythm diagnosis
    - defibrillate in under 10 seconds from rhythm diagnosis
    - resume chest compressions immediately after defibrillation

Materials needed for this scenario:

- RessusciAnne® simulator with remote control
- Zoll R series® defibrillator
- Bag-valve-mask device with reservoir
- Laryngoscope
- Endotracheal tubes (size 7.0, 7.5 and 8.0)
